# Compositional Influence on the Morphology and Thermal Properties of Woven Non-Woven Mats of PLA/OLA/MgO Electrospun Fibers

**DOI:** 10.3390/polym14102092

**Published:** 2022-05-20

**Authors:** Adrián Leonés, Laura Peponi, Jesús-María García-Martínez, Emilia P. Collar

**Affiliations:** 1 Instituto de Ciencia y Tecnología de Polímeros (ICTP-CSIC), C/Juan de la Cierva 3, 28006 Madrid, Spain; aleones@ictp.csic.es (A.L.); jesus.maria@ictp.csic.es (J.-M.G.-M.); ecollar@ictp.csic.es (E.P.C.); 2 Interdisciplinary Platform for “Sustainable Plastics towards a Circular Economy” (SUSPLAST-CSIC), 28006 Madrid, Spain

**Keywords:** electrospinning, poly(lactic acid), magnesium oxide, oligomer(lactic acid), design of experiments

## Abstract

In the present work, a statistical study of the morphology and thermal behavior of poly(lactic acid) (PLA)/oligomer(lactic acid) (OLA)/magnesium oxide nanoparticles (MgO), electrospun fibers (efibers) has been carried out. The addition of both, OLA and MgO, is expected to modify the final properties of the electrospun PLA-based nanocomposites for their potential use in biomedical applications. Looking for the compositional optimization of these materials, a Box–Wilson design of experiment was used, taking as dependent variables the average fiber diameter as the representative of the fiber morphologies, as well as the glass transition temperature (T_g_) and the degree of crystallinity (X_c_) as their thermal response. The results show <r^2^> values of 73.76% (diameter), 88.59% (T_g_) and 75.61% (X_c_) for each polynomial fit, indicating a good correlation between both OLA and MgO, along with the morphological as well as the thermal behavior of the PLA-based efibers in the experimental space scanned.

## 1. Introduction

Poly(lactic acid), PLA, is one of the most studied biobased polymers, not only for its biodegradability and renewable source [1], but also for its easy processability [2], low weight [3] and transparency [4]. However, its brittle nature and its low mechanical properties in terms of elongation at break and tensile strength are still drawbacks that need to be improved [5]. Otherwise, PLA shows degradability under physiological conditions into non-toxic products, making it an ideal material to be used in contact with the human body [6,7]. However, considering some biomedical applications, such as films or woven/non-woven electrospun fibers with thermally-activated shape memory behavior for uses in contact with human tissues, its glass transition temperature, T_g_, (60 °C) is much higher than the human body temperature, which needs to be tailored [8].

Nowadays, the use of both plasticizers and nanoparticles, NPs, are widely used in order to enhance the mechanical response and modulate the thermal properties of PLA [9,10]. In general, the addition of new components into a polymer strongly affected the final reinforced polymeric matrix behavior [11]; therefore, their interactions have to be studied to guarantee the optimal performance of the material [12]. Moreover, the effect of the different components on the properties of polymer nanocomposites depends on several factors, such as the complete dispersion of the nanoparticles in the matrix and the consequent development of a huge interfacial area [9,11,13,14].

With this background, the ideal plasticizer should have a similar chemical structure to the matrix and appropriate molecular weight to assure the good compatibility between the matrix and the NPs, therefore, enhancing the final thermal and mechanical properties of the nanocomposites [14,15]. In particular for PLA matrix, several additives have been tested as potential plasticizers, such as glycerol [16], poly(ethylene glycol) [17], acetyl(tributyl citrate) [18] or glyceril triacetate [19]. In recent years, an oligomer of lactic acid, OLA, has been proposed as an alternative to common plasticizers for PLA, taking advantage of its similar chemical structure [20,21,22,23]. Some studies can be found in the literature in this way. In particular, Cicogna et al. [21] reported the structural, thermal and biodegradability properties of PLA films plasticized with OLA. Additionally, Burgos et al. [20] studied different PLA films plasticized with three OLAs synthesized under different conditions and reported enhanced mechanical properties in terms of elongation at break. The use of OLA as a plasticizer for PLA matrix is not limited to films. In fact, the electrospinning process can be used to obtain PLA plasticized electrospun fibers, efibers. The electrospinning process is the most suitable technique used to produce a wide variety of woven non-woven mats starting from a polymeric solution exposed to high electric fields [24]. Moreover, it is one of the most efficient, simple and versatile processing techniques able to produce fibers for different applications, such as drug delivery [25], food packaging [26], or tissue engineering [27]. The use of OLA as a plasticizer in PLA-based efibers has been recently studied and reported in the literature [8,26,28]. In particular, Leonés et al. [8] obtained PLA-based efibers plasticized with OLA at different concentrations and studied its thermally-activated shape memory behavior tailoring their T_g_ to a temperature close to the physiological one. In another paper regarding biomedical applications, the in vitro degradation of PLA-based efibers plasticized with OLA has been reported in simulated body fluid until reaching the complete degradation of the samples [28]. They found that the addition of OLA increases the hydrolytic degradation process of PLA-based fiber mats. Moreover, by adding different amounts of OLA, such as 10, 20 and 30 wt%, the time of degradation in phosphate buffered saline, PBS, can be modulated over the course of a year.

On the other hand, the dispersion of different NPs through efibers has been studied with very promising results in different fields, such as food packaging [29], environmental, agricultural [30], and biomedical applications [31]. In particular, in the biomedical field, the main purpose is to produce scaffolds that guide the growth of cells and temporarily serve as a support for cell attachment and differentiation [10]. Thus, scaffolds have to successfully mimic the natural chemical and biological environment and the mechanical properties of the injured tissue [10]. With this aim, different inorganic NPs have been studied to obtain woven non-woven electrospun mats with specific requirements, such as porosity [32], biocompatibility [33], or enhanced mechanical properties [27,34]. Among the inorganic NPs, magnesium oxide nanoparticles, MgO NPs, have been recently studied as reinforcement in polymer matrices due to the role of magnesium in cellular functions, its good biocompatibility and its crucial role in bone growth [10,35]; thus MgO NPs have emerged in recent years as promising inorganic nanofillers for biopolymers [10]. Some authors have recently reported the improved mechanical properties of polymeric efibers reinforced with MgO NPs. For instance, Boakye et al. reported a slight increase in the mechanical properties with the addition of MgO NPs to poly(ɛ-caprolactone)-keratin efibers [36]. De Silva et al. studied alginate-based efibers reinforced with MgO NPs and reported enhanced tensile strength and elastic modulus [37].

However, no works can be found in the scientific literature about PLA-based efibers with both OLA as a plasticizer and MgO NPs.

Moreover, nowadays, the results of the electrospinning process are still partially unpredictable and exhibit a varying average diameter of efibers which limits its usefulness in industrial applications [38]. At this point, it is important to study the average diameter of the efibers in order to increase the validity and repeatability of the process [39]. In addition, the average diameter is influenced by different parameters involved during the electrospinning process, such as the applied voltage, flow rate, or polymer concentration [10]. It is, therefore, critical to analyze this parameter as representative of the processing of the electrospun fibers.

Therefore, in this work, a preliminary statistical study of the components and their interactions has been carried out in order to study their effects on the PLA matrix. In particular, the main goal of the present study is the optimization of the thermal properties within the experimental range of the composition OLA and MgO for the PLA-based efibers. For this purpose, it is very important to use a response surface methodology, RSM, able to fit any given dependent variables to polynomial equations over the experimental range scanned as a function of those considered as independent ones [40,41]. The RSM encompasses the use of different types of designs of experiments (DOE) which reduce the number of experimental runs necessary to set a reliable mathematical trend within the experimental space scanned for a given response [42,43]. The obtained correlations can be used in further optimization steps. When the limits of the main independent variables are known, the Box–Wilson RSM is one of the most suitable tools for optimization purposes. The Box–Wilson RSM is a central rotatory composite design with (2*^k^* + 2*k* + 1) experiments, plus (2 + *k*) replicated central runs, where k is the number of independent variables [42,43]. Based on previous studies [8,28] a Box–Wilson RSM has been carried out with two independent variables, the amount of OLA and of MgO, considered as the responses to both the morphology and the thermal properties of the efibers. For the former, the evolution of the fiber diameters was chosen, while for the latter they were the glass transition temperature and the degree of crystallinity of the PLA polymer matrix in the nanocomposites. As we will see, very interesting results emerging from the present work include that not only do the two components play an important role in the evolution of the properties of the fiber but there is also a crossed effect linked to the processing step and related to the ratio between both the MgO and the OLA amounts.

## 2. Materials and Methods

Polylactic acid (PLA3051D), 3% of D-lactic acid monomer, molecular weight 14.2 × 10^4^ g·mol^−1^, density 1.24 g·cm^−3^) was supplied by NatureWorks^®^ (NatureWorks LLC, Minnetonka, MN, USA). Lactic acid oligomer (Glyplast OLA8, ester content > 99%, density 1.11 g·cm^−3^, viscosity 22.5 mPa·s, molecular weight 1100 g·mol^−1^) was kindly supplied by Condensia Quimica SA (Barcelona, Spain). Chloroform, CHCl_3_, (99.6% purity) and *N,N*-dimethylformamide, DMF, (99.5% purity) from Sigma Aldrich (Madrid, Spain) were used as solvents. Magnesium oxide nanoparticles (MgO NPs, average particle size of 20 nm, 99.9% purity, molecular weight 40.30 g∙mol^−1^) were supplied by Nanoshel LLC (Wilmington, Delaware, USA).

Previous to the electrospinning process, each solution was prepared following the following steps. Firstly, the corresponding amounts of PLA and OLA were dissolved separately in CHCl_3_ and stirred overnight at room temperature. Secondly, the amount of MgO NPs was weighed and dispersed in 20 mL of CHCl_3_; after 30 min the OLA solution was added and dispersed for 60 min. Then, the PLA solution was added and dispersed for another 60 min. Finally, we added the necessary volume of DMF to assure the proportion of solvents CHCl_3_:DMF (4:1). The dispersion process was carried out with a sonicator tip (Sonic Vibra-Cell VCX 750, Sonics & Materials, Newton, CT, USA) of 750 watts and an amplitude of 20%. Then, electrospun fiber mats were obtained in an Electrospinner Y-flow 2.2.D-XXX (Nanotechnology Solutions, Malaga, Spain) in vertical configuration coupled to coaxial concentric needles. Polymer solutions were pumped through the inner needle and a CHCl_3_:DMF (4:1) solvent solution was pumped through the outer needle. An electric field of 10 kV in positive and −10 kV in negative poles was set. The flow rates for both the solvent and the polymer solution were fixed at 0.50 mL∙h^−1^ and 3.5 mL∙h^−1^, respectively. Each formulation was electrospun for 3 h over a metal plane collector covered with aluminum foil placed at a 14 cm distance from the needle. The obtained mats were vacuum dried for 24 h in order to remove any solvent residues [8].

The different amounts of OLA and MgO studied were set according to the specifications of the Box–Wilson experimental worksheet (Table 1). The range of the independent variable OLA was set from 6.00 to 30.00 wt% while for MgO it was from 0.60 to 3.00 wt% being, respectively, coded as the lowest and the highest (−1, 1). Additionally, the Box–Wilson experimental model considers α = √2 as the coded variable for the star points of the worksheet [44,45]. Therefore, in Table 1 all the coded and the controlled factors are listed together with each run number. The corresponding trials were conducted in a randomized way. The variables were statistically analyzed by one-way analysis of variance (ANOVA) and using the statistical computer package Statgraphics Centurion XVII (Statpoint Technologies, Inc., Warrenton,, VA, USA) [44].

Scanning Electron Microscopy, SEM, PHILIPS XL30 Scanning Electron Microscope, (Phillips, Eindhoven, The Netherlands) was used in order to study the morphology of the efibers. All the samples were previously gold-coated (~5 nm thickness) in a Polaron SC7640 Auto/Manual Sputter (Polaron, Newhaven, East Sussex, UK). SEM image analyses were carried out with ImageJ software (Bethesda, Maryland, USA). Diameters were calculated as the average value of 30 random measurements for each sample.

Thermal transitions were studied by Differential Scanning Calorimetry, DSC, in a DSC Q2000 TA instrument under a nitrogen atmosphere (50 mL∙min^−1^). The samples were cooled from room temperature to −60 °C at 20 °C∙min^−1^, then, the thermal analysis was programmed at 10 °C∙min^−1^ from −60 °C up to 180 °C obtaining the glass transition temperature (T_g_) calculated as the midpoint of the transition, the cold crystallization enthalpy (ΔH_cc_) and the melting enthalpy (ΔH_m_). The degree of crystallinity (X_c_%) was calculated using Equation (1), taking the value of crystallization enthalpy of pure crystalline PLA (ΔH_m_°) as 93.6 J∙g^−1^ and W_f_ as the weight fraction of PLA in the sample [45].

(1) $$X_{c}\left(\%\right)=\frac{{\Delta H}_{m}-{\;\Delta H}_{\mathrm{cc}}\;}{{\Delta H}_{m}{}^{\circ}}\;\times \;\frac{1}{W_{f}}\;\times \;100$$

## 3. Results and Discussion

**Figure 1:**
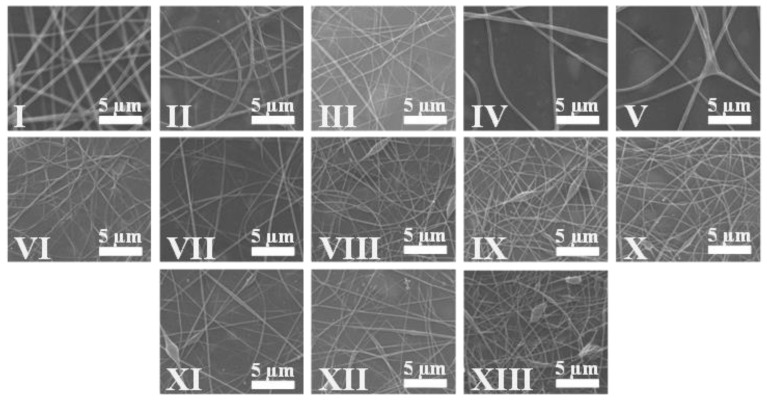
SEM images of the different electrospun mats obtained from run I to run XIII of the experimental worksheet in Table 1.

Once we obtained the different woven non-woven electrospun mats for each run, their fiber morphologies were studied by SEM images. Figure 1 displays typical SEM images of the woven non-woven mats from each one of the runs of Table 1, representing the experimental worksheet. As can be seen, straight and randomly oriented fibers with different average diameters, reported in Table 2, were obtained for each run, which indicates the suitability of the experimental range chosen by using experimental conditions previously reported by us [8,28]. Some beads, small in size, may be observed in the different samples and are considered typical defects when processing by electrospinning, and are associated with solution viscosity and increments in the surface tension [46].

It is possible to note the differences in the diameter between the run V and the run VI SEM images, which are corresponding, respectively, to the lowest and the highest OLA amount in the nanocomposites, and both contain 1.8 wt% of MgO NPs. From a qualitative point of view, the lower diameters in run VI compared to those in run V, agree with the decrease in the viscosity values of the run VI solutions of 35 wt% OLA can be expected, compared to the run V solution with has just 1.03 wt% of OLA.

**Figure 2:**
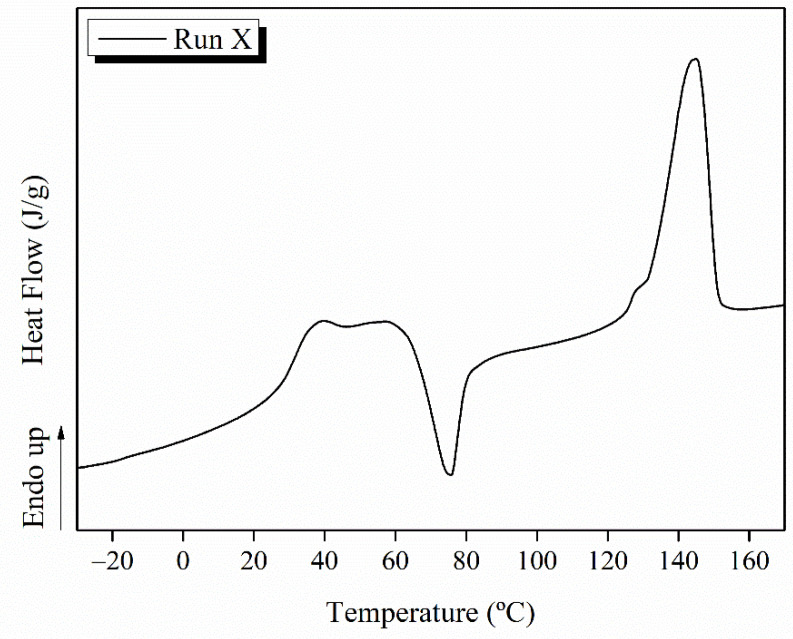
DSC thermogram for PLA-based efibers obtained in run X.

The quantitative dimension of the morphological study will be given by the polynomial fit to surface response of the average fiber diameter values reported in Table 2, which indicates the glass transition temperature, T_g_ and the degree of crystallinity, X_c_, of PLA, values obtained from DSC thermograms for the different nanocomposites obtained, according to the Box–Wilson worksheet. Moreover, an example of a characteristic DSC thermogram of PLA-based efibers is shown in Figure 2.

As can be seen in Table 2, all electrospun nanocomposites show a higher degree of crystallinity than the value obtained for the neat PLA efibers, X_c_ = 1.2%. In particular, runs II, IV and VI show the lowest T_g_ and are the samples with the highest values for X_c_. Run II shows a T_g_ of 31 °C and X_c_ of 30%, run IV a T_g_ of 32 °C and 17.6% and run VI a T_g_ of 34 °C and X_c_ of 16.6%, as we expect, lower T_g_ indicates major mobility of the polymeric chains and as consequence, better facility to crystallize and therefore higher X_c_ values.

The diameter, T_g_ and X_c_ of the PLA nanocomposites were fitted to quadratic models by following the Box–Wilson response surface methodology RSM [42,43]. Therefore, three different polynomials with quadratic and interaction terms were properly obtained having the general form:

Y = a_0_ + a_1_∙x_1_ + a_2_∙x_2_ + a_3_∙x_1_∙x_2_ + a_4_∙x_1_^2^ + a_5_∙x_2_^2^

The coefficients obtained for each polynomial fit are reported in Table 3, together with the percentual confidence values for <r^2^>, the lack of fit and the confidence factors coefficients, obtained from the ANOVA, which informs about the accuracy and significance of the variables.

The <r^2^> (%) value is the first indicator of how well a model fits a data set and, accordingly, how well a model can predict the value of the response of the variable studied in percentage terms. As reported in the literature [42,43], <r^2^> values above 70.00% are considered good fitting for quadratic models. In our case, the <r^2^> values obtained were 73.76% for diameter, 88.59% for the T_g_ and 75.61% for X_c_. Therefore, it indicates the significance of OLA and MgO NPs content as the independent variables chosen to model the diameter, T_g_ and X_c_ of the PLA-based efibers in our experimental range studied.

Table 3 also shows the lack of fit values associated with the percentage of pure error. In fact, the lack of fit values tells us about possible factors overlooked by the model or a poor choice of variables, but significant in the response development. High values of lack of fit indicate that this parameter is more sensitive to the noise effects of the experiment carried out. As can be seen, lack of fit values of 1.5%, 5.2% and 18.9% were obtained for diameter, T_g_ and X_c_, respectively.

Additionally, high values for the confident factors indicate the full significance of the independent variables chosen in this study. In fact, in our system, confidence factors of 94.1%, 99.1% and 95.1% for diameter, T_g_ and X_c_, respectively, indicate that all the factors considered to build our model play a prime role in the behavior of PLA-based efibers. Consequently, all the parameters summarized in Table 3 confirm the successful choice of studying the system from the Box–Wilson model forecast.

Firstly, the limitations of the model, if any, have to be checked. In this regard, the predicted versus the plot of the measured value is one of the most common alternatives to evaluate models by studying the scatter in a set of data [47].

**Figure 3:**
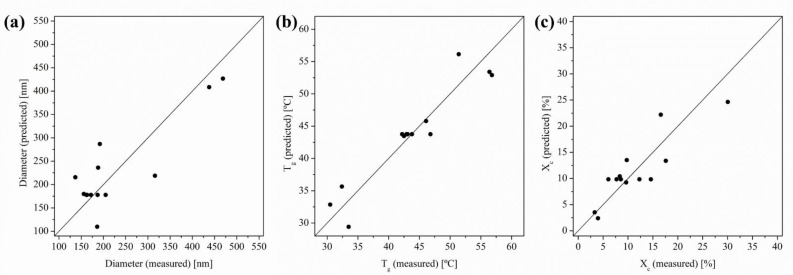
Predicted versus measured values for (**a**) diameter, (**b**) glass transition temperature and (**c**) degree of crystallinity.

In Figure 3, in order to explore this aspect, the predicted versus the measured values for diameter, T_g_ and X_c_, are plotted, respectively, in Figure 3a–c. A very good correlation between measured and predicted values and a good scatter of the set of data for each property was observed indicating homoscedastic distribution.

Additionally, Table 4 compiles the confidence coefficient (%) and *t*-value for the different terms of each polynomial equation of the Box–Wilson model obtained for the studied properties. In general, the higher the confidence coefficient, the more certain are the results. On the other hand, the *t*-value measures how many standard errors the coefficients are from zero. Generally, any *t*-value higher than 2 is significant and the higher the *t*-value for a term, the greater the confidence in this term [42,43].

We can remark that the diameters show highly significant t-values for both the linear and quadratic terms in the case of OLA which are 3.33 and 2.61, respectively, with very high confidence coefficients, showing a high dependence of this property on OLA content. Additionally, the *t*-value in the limit of significance for the linear parameter in the case of MgO NPs content, 1.54, indicates a slight dependence of diameter on the MgO NPs content. This is also confirmed by its confidence coefficients. A different case relates to the glass transition temperature where only the linear term in the case of OLA, 1.72, can be considered in the limit of significance revealing a dependence of T_g_ on the plasticizer content. No dependence of T_g_ on MgO NPs content is significantly observed. In the case of X_c_ neither the t-values for the linear term, nor for the quadratic terms indicate its dependence on both OLA and MgO content, while the t-value for its interaction terms, 1.96, proves the significance of the interfacial interaction between the plasticizer and the nanoparticles. Scientific implications of these comments will be discussed under the next points, over the corresponding dependent vs independent variable plots.

### 3.1. Influence of OLA and MgO NPs Content in the Average Diameter of the PLA-Based Efibers

**Figure 4:**
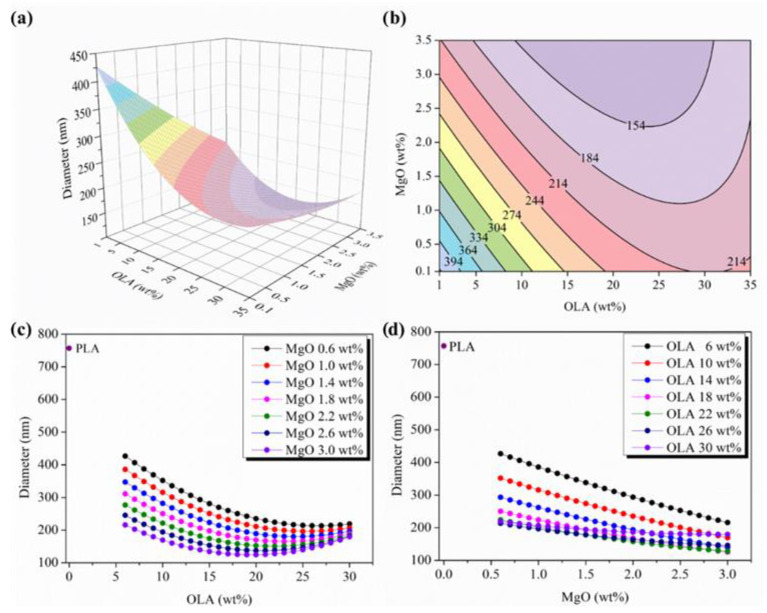
(**a**) 3D response surface plot and (**b**) contour plot of the diameter as a function of OLA and MgO NPs content, colours changes are attributed to an increment of 30 nm in the average diameter of efibers. (**c**) Parametric evolution of diameter with OLA content remaining constant the MgO NPs levels. (**d**) Parametric evolution of diameter with MgO NPs content remaining constant the OLA amounts.

The changes observed in the average diameter evolution in terms of OLA and MgO NPs content have been plotted in Figure 4. In particular, Figure 4a shows the 3D response surface plot for the diameter and Figure 4b shows the contour map properly explaining the evolution of the diameter as a complex function of both OLA and MgO NPs content. First of all, it can be observed that neat PLA efibers show the highest values of diameters with respect to those efibers obtained when both OLA and MgO NPs have been added at different concentrations.

As described above, the average diameter of the efiber mats is considered representative of studying the processing of the fiber. In general, nanofiller additions tend to increase the viscosity of electrospun polymer solutions [10,48]. However, if nanofillers are conducting materials, such as MgO NPs [49,50], they can strengthen the repulsive force generated during the electrospinning process by increasing the solution conductivity and hence decreasing the fiber diameters. Thus, there is a balance between the viscosity and repulsive force in forming electrospun mats during electrospinning [48].

The parametric plots showing, respectively, the evolution of diameters with OLA at constant levels of MgO NPs, and with the MgO NPs content at constant levels of OLA, have been included in Figure 4c,d. The addition of both, OLA and MgO NPs to PLA, provokes a decrease in the average diameter of the efibers with respect to neat PLA efibers. However, a different evolution of the average diameter can be observed for each independent variable, OLA and MgO NPs. In Figure 4c, the parametric evolution of diameter versus OLA content shows a similar pattern of hyperbola where diameter decreases by increasing the amount of OLA, as expected. However, above 15 wt% of OLA, there is smoothing in the slopes of the hyperbolic curve that reaches up to 25 wt%, where the minimum average diameters can be observed. Finally, from 25 wt% of OLA, the average diameter increases and almost converges at the same value, meaning that at this point, the relation between the increase in viscosity due to the increasing content of MgO NPs is no longer compensated by the increase in the conductivity of the solutions due to the presence of the MgO NPs [47]. Between 17 wt% and 22 wt% of OLA, the effect on the viscosity and on the conductivity due to MgO NPs seems to yield the lowest average diameter of the efibers.

### 3.2. Influence of OLA and MgO NPs Content in the Glass Transition Temperature of the PLA-Based Efibers

**Figure 5:**
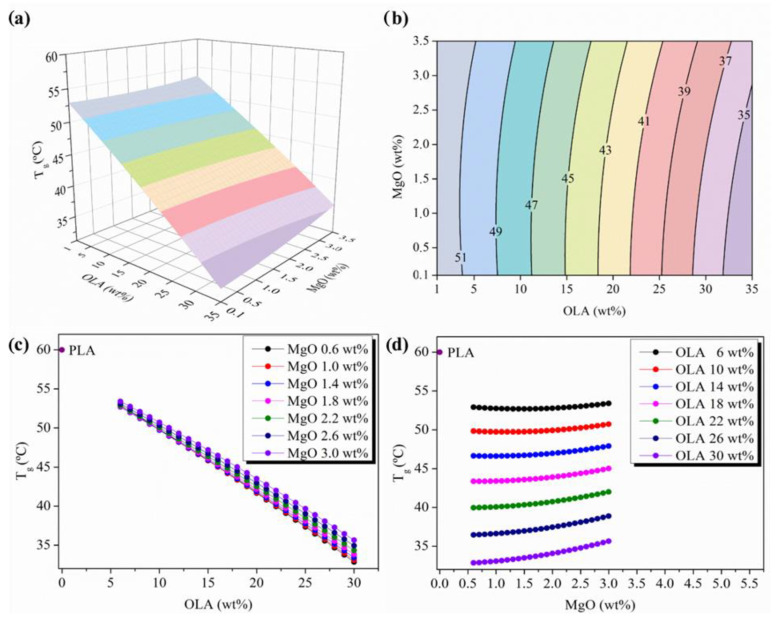
(**a**) 3D response surface plot and (**b**) contour plot of the glass transition temperature as a function of OLA and MgO NPs content, colours changes are attributed to an increment of 2 °C in the T_g_. (**c**) Parametric evolution of glass transition temperature with OLA content for different amounts of MgO NPs levels. (**d**) Parametric evolution of glass transition temperature with MgO NPs content for different amounts of OLA amounts.

The changes observed in the T_g_ evolution have been plotted in Figure 5. In particular, Figure 5a shows the 3D response surface plot for T_g_ and Figure 5b the contour map explaining the evolution of the T_g_ as a complex function of both OLA and MgO NPs content. Firstly, it is proper to remark that the T_g_ isolines follow an almost parallel evolution and are hardly influenced by the MgO NPs content up to 2 wt%. In particular, a decrease from 9 to 25 °C by increasing the OLA content in comparison with the T_g_ of neat PLA (60 °C) in the experimental space scanned, evidenced that the saturation in OLA content is not reached. This behavior is clearly observed in Figure 5c,d, where the parametric evolution of T_g_ versus OLA and MgO NPs content is shown, respectively.

The parametric evolution of T_g_ versus OLA content (Figure 5c) evidences the almost linear decreasing dependency of this parameter as the amount of OLA increases, with overlapping for almost all the amount of MgO NPs. In fact, neat PLA efibers show a T_g_ of 60 °C and the addition of the minimum amount of OLA studied, that is 6 wt%, decreased the T_g_ value to about 53 °C for both the minimum and maximum amount of MgO NPs studied, that is, 0.6 and 3.0 wt%, respectively. This behavior is observed in all the space scanned until the region of 22 wt% OLA, from this point the isolines begin to separate slightly from each other reaching the maximum amount of OLA studied, that is, 30 wt%, and the distance from each isoline is the highest observed. From the other side, a slight variation in the T_g_ values can be observed in Figure 5c between the highest and the lowest amount of MgO NPs. In fact, at 30 wt% OLA, when 0.6 MgO wt% has been added, the T_g_ is 32 °C while when 3 MgO wt% is added, the T_g_ increases to 35 °C, about 10%. This is a very interesting result that takes into account that usually the addition of NPs at above a minimal concentration leads to an increase in the T_g_ value of nanocomposites. In particular, Khan et al. reported that at a high volume fraction of NPs, the T_g_ shows an increasing trend, with an increase in the amount of NPs due to the higher number of contacted polymer chains with NPs and the diffusion of NPs. They concluded that the T_g_ can be controlled by the agglomeration of NPs and the interaction strength between the NPs and polymer chains [51,52]. In our case, this concentration is not reached in the experimental space scanned and the T_g_ is only affected by the addition of OLA.

Therefore, as can be seen in Figure 5c, the T_g_ of the nanocomposites decreases almost linearly as the OLA content increases, going into the right physiological temperatures window once the 20 wt% OLA content is reached. From here, the overlap observed in the MgO NPs isolines disappears, with those with the highest levels of MgO NPs, 3 wt%, showing the highest T_g_. The effect is clearly seen in Figure 5d, where the T_g_ values evolve almost parallel and equidistant from each other for the lowest OLA and MgO NPs content. A slight increase in the T_g_ of PLA-reinforced efibers from the values of MgO NPs that are higher than 2.0 wt% is observed.

### 3.3. Influence of OLA and MgO NPs Content in the Degree of Crystallinity of the PLA-Based Efibers

**Figure 6:**
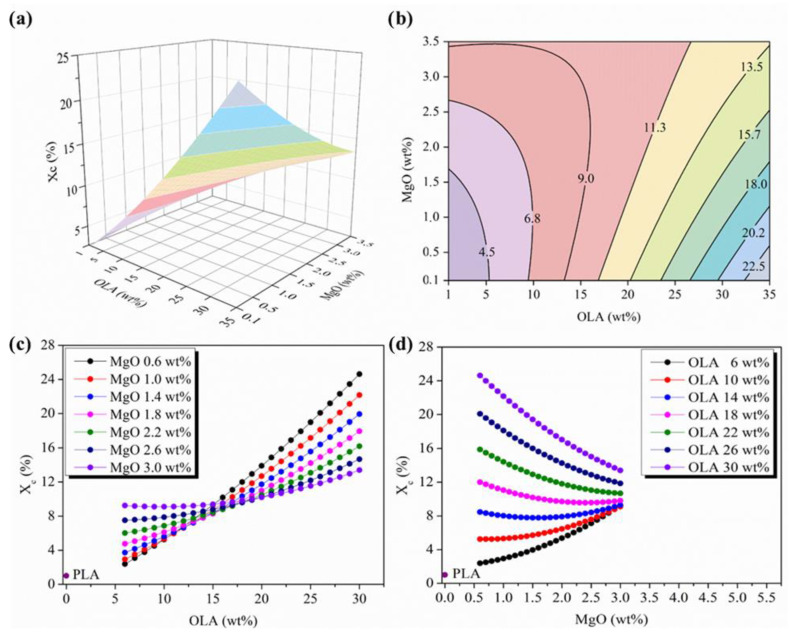
(**a**) Three-dimensional response surface plot and (**b**) contour plot of the degree of crystallinity as a function of OLA and MgO NPs content, colours changes are attributed to an increment of 2.3 % in X_c_. (**c**) Parametric evolution of the degree of crystallinity with OLA content for different amounts of MgO NPs levels. (**d**) Parametric evolution of the degree of crystallinity with MgO NPs content for different amounts of OLA amounts.

As described in the literature, the addition of NPs into polymeric matrices is expected to increase the degree of crystallinity due to the nucleation effect of the nanofillers [27,53]. Moreover, some authors have previously reported the capability of OLA to crystallize within the PLA matrix when processed by electrospinning [8,23,28]. Thus, when working with inorganic NPs and OLA plasticizers in PLA-based efibers, the effect of both additives in the X_c_ has to be considered. In order to study the evolution of this parameter in the experimental space scanned, the response surface plot and the contour map of the degree of crystallinity, calculated by DSC, as a function of OLA and MgO NPs content is reported in Figure 6a,b.

As can be observed in Figure 6c, the degree of crystallinity of PLA-based efibers linearly increases with the amount of OLA, showing the highest slopes in the isolines for the lower amount of MgO NPs. This slope progressively smoothens by increasing the amount of MgO NPs until a constant degree of crystallinity of 10% is observed for the highest amount of NPs, that is, MgO NPs 3 wt% (purple isoline in Figure 6c). Moreover, when the amount of OLA reaches 15 wt%, the convergence of the degree of crystallinity takes place for all the MgO NPs content. In particular, from OLA 15 wt%, the degree of crystallinity linearly increases by increasing the amount of OLA with higher slopes for those isolines related to the lower amount of MgO NPs. As can be observed, the highest value of the degree of crystallinity, X_c_ = 24%, corresponds to the maximum amount of OLA, 30 wt%, and the lowest amount of MgO NPs, which is 0.6 wt%. In fact, it is worth noting, that by increasing the amount of MgO NPs, for the same OLA concentration of 30 wt%, the X_c_ decreases.

The representation of X_c_ at the constant levels of OLA shown in Figure 6d shows the same behavior. The highest degrees of crystallinity are observed for the highest amounts of OLA. Moreover, the X_c_ isolines decrease by increasing the MgO NPs content, smoothing progress on the slopes until reaching the isoline of 18 wt% OLA (pink isoline in Figure 6d). Above 2 wt% MgO NPs, the inversion in the direction of the slopes occurs, which becomes positive for the lower amounts of OLA, showing a degree of crystallinity values towards X_c_ = 10%.

Some authors previously studied the synergic effects between other nucleating agents and plasticizers, even at very small concentrations, on the degree of crystallinity of PLA nanocomposites, suggesting that, given the enhanced mobility of plasticized PLA, very small concentrations of NPs are effective nucleation agents. They reported a critical amount where a strong effect on the crystallization process was observed and which increases the mechanical properties [54,55]. In our PLA-based efibers, the detection of a region of convergence in a certain region of OLA and MgO NPs content, is coincident with those previously detected for the evolution of the glass transition temperature and diameter, again suggesting a critical MgO/OLA ratio related to the processing stage and determinant in the behavior characteristics of the nanocomposites. The mechanical behavior of these PLA-based efibers will be discussed in upcoming studies, as well as in subsequent studies regarding the design of the experimentation.

## 4. Conclusions

The role of the addition of both OLA as a plasticizer and MgO as a nanofiller into electrospun PLA fibers has been determined by studying the average diameter of fibers, their glass transition temperature and the degree of crystallinity by statistical analysis. In our electrospun nanocomposites, the interactions between the components determine the final properties and a Box–Wilson model has been used in order to identify the level of interactions and to look for the optimal compositional ratios. The <r^2^> values obtained were 73.76% (diameter), 88.59% (T_g_) and 75.61% (X_c_), respectively, and considered good fitting for quadratic models. In consequence, the predicted versus the measured values for diameter, T_g_ and X_c_, showed a very good correlation evolving as homoscedastic distributions.

The interval of OLA content which leads to a minimum average diameter of fibers has been identified; the results obtained for the glass transition temperature evolution evidence a clear dependency of this parameter on the amount of OLA, whose parametric isolines are almost overlapping for all the amount of MgO NPs. Finally, the synergic effect of OLA and MgO NPs in the degree of crystallinity of the PLA-based efibers increases this parameter from almost zero in the pristine PLA efibers to a range values from 2–24%. A critical point in the X_c_ evolution has been identified whose coordinates are fully coincident with the critical ones for the other studied properties.

## Figures and Tables

**Table 1:** Worksheet for the Box–Wilson experimental design used.

Run	Coded Factors		Controlled Factors	
	OLA	MgO	OLA (wt%)	MgO (wt%)
I	−1	−1	6.00	0.60
II	1	−1	30.00	0.60
III	−1	1	6.00	3.00
IV	1	1	30.00	3.00
V	−√2	0	1.03	1.80
VI	√2	0	34.97	1.80
VII	0	−√2	18.00	0.10
VIII	0	√2	18.00	3.49
IX	0	0	18.00	1.80
X	0	0	18.00	1.80
XI	0	0	18.00	1.80
XII	0	0	18.00	1.80
XIII	0	0	18.00	1.80

**Table 2:** Efibers experimental responses: Average diameter values, glass transition temperature, T_g_, and degree of crystallinity, X_c_.

Run	Diameter (nm)	T_g_ (°C)	X_c_ (%)
I	469 ± 106	57	3.9
II	316 ± 67	31	30.0
III	137 ± 16	56	9.6
IV	156 ± 38	32	17.6
V	438 ± 67	51	3.3
VI	188 ± 45	34	16.6
VII	192 ± 39	43	9.8
VIII	186 ± 56	46	8.4
IX	172 ± 35	42	6.1
X	187 ± 43	44	14.6
XI	205 ± 62	43	12.3
XII	162 ± 42	47	7.7
XIII	163 ± 36	43	8.6

**Table 3:** Statistical parameters and coefficients of the polynomial equations from the Box–Wilson experimental design used. (Y = a_0_ + a_1_∙x_1_ + a_2_∙x_2_ + a_3_∙x_1_∙x_2_ + a_4_∙x_1_^2^ + a_5_∙x_2_^2^).

	<r^2^> (%)	L. F. * (%)	C. F. * (%)	Ind. T. *	L. T. *		Int. T. *	Q. T. *	
				a_0_	a_1_	a_2_	a_3_	a_4_	a_5_
Diameter (nm)	73.76	1.5	94.1	645.3	−28.51	−131.4	2.986	0.5016	7.101
T_g_ (°C)	88.59	5.2	99.1	57.86	−0.7364	−1.113	0.03993	−0.003420	0.3004
X_c_ (%)	75.61	18.9	95.1	−2.816	0.7388	2.096	−0.3142	0.01046	0.7352

* L.F. (Lack of fit), C. F. (Confident Factor), Ind. T. (Independent Term), L. T. (Linear Terms), Int. T. (Interaction Term), Q. T. (Quadratic Terms).

**Table 4:** *t*-value and confidence coefficient (%) for the different terms of each polynomial equations of the Box–Wilson model obtained for the studied properties.

	Ind. T. *	L. T. *		Int. T. *	Q. T. *	
		x_1_	x_2_	x_1_·x_2_	x_1_^2^	x_2_^2^
Diameter (nm)	5.31 (99.7%)	3.33 (98.6%)	1.54 (82.8%)	1.18 (71.0%)	2.61 (96.6%)	0.37 (31.1%)
T_g_ (°C)	9.51 (99.9%)	1.72 (87.0%)	0.26 (26.0%)	0.31 (28.5%)	0.36 (30.5%)	0.31 (28.4%)
X_c_ (%)	0.37 (31.0%)	1.37 (77.8%)	0.39 (32.0%)	1.96 (91.1%)	0.86 (56.6%)	0.61 (43.3%)

* Ind. T. (Independent Term), L. T. (Linear Terms), Int. T (Interaction Term), Q. T. (Quadratic Terms).

## Data Availability

Not applicable.

## References

[1] Singhvi M., Gokhale D. (2013). Biomass to biodegradable polymer (PLA). RSC Adv..

[2] Nofar M., Sacligil D., Carreau P.J., Kamal M.R., Heuzey M.-C. (2019). Poly (lactic acid) blends: Processing, properties and applications. Int. J. Biol. Macromol..

[3] Rhim J.-W., Park H.-M., Ha C.-S. (2013). Bio-nanocomposites for food packaging applications. Prog. Polym. Sci..

[4] Raquez J.M., Habibi Y., Murariu M., Dubois P. (2013). Polylactide (PLA)-based nanocomposites. Prog. Polym. Sci..

[5] Farah S., Anderson D.G., Langer R. (2016). Physical and mechanical properties of PLA, and their functions in widespread applications—A comprehensive review. Adv. Drug Deliv. Rev..

[6] da Silva D., Kaduri M., Poley M., Adir O., Krinsky N., Shainsky-Roitman J., Schroeder A. (2018). Biocompatibility, biodegradation and excretion of polylactic acid (PLA) in medical implants and theranostic systems. Chem. Eng. J..

[7] Zaaba N.F., Jaafar M. (2020). A review on degradation mechanisms of polylactic acid: Hydrolytic, photodegradative, microbial, and enzymatic degradation. Polym. Eng. Sci..

[8] Leonés A., Sonseca A., López D., Fiori S., Peponi L. (2019). Shape memory effect on electrospun PLA-based fibers tailoring their thermal response. Eur. Polym. J..

[9] Darie-Niţă R.N., Vasile C., Irimia A., Lipşa R., Râpă M. (2016). Evaluation of some eco-friendly plasticizers for PLA films processing. J. Appl. Polym. Sci..

[10] Leonés A., Lieblich M., Benavente R., Gonzalez J.L., Peponi L. (2020). Potential applications of magnesium-based polymeric nanocomposites obtained by electrospinning technique. Nanomaterials.

[11] Peponi L., Puglia D., Torre L., Valentini L., Kenny J.M. (2014). Processing of nanostructured polymers and advanced polymeric based nanocomposites. Mater. Sci. Eng. R. Rep..

[12] García-Martínez J.M., Areso S., Collar E.P. (2009). The role of a novel p-phenylen-bis-maleamic acid grafted atactic polypropylene interfacial modifier in polypropylene/mica composites as evidenced by tensile properties. J. Appl. Polym. Sci..

[13] Nofar M., Salehiyan R., Ray S.S. (2021). Influence of nanoparticles and their selective localization on the structure and properties of polylactide-based blend nanocomposites. Compos. Part B Eng..

[14] Rahman M., Brazel C.S. (2004). The plasticizer market: An assessment of traditional plasticizers and research trends to meet new challenges. Prog. Polym. Sci..

[15] Bocqué M., Voirin C., Lapinte V., Caillol S., Robin J.-J. (2016). Petro-based and bio-based plasticizers: Chemical structures to plasticizing properties. J. Polym. Sci. Part A Polym. Chem..

[16] Satriyatama A., Rochman V.A.A., Adhi R.E. (2021). Study of the Effect of Glycerol Plasticizer on the Properties of PLA/Wheat Bran Polymer Blends. IOP Conf. Ser. Mater. Sci. Eng..

[17] Pivsa-Art W., Fujii K., Nomura K., Aso Y., Ohara H., Yamane H. (2016). The effect of poly(ethylene glycol) as plasticizer in blends of poly(lactic acid) and poly(butylene succinate). J. Appl. Polym. Sci..

[18] Arrieta M.P., Peponi L., López D., Fernández-García M. (2018). Recovery of yerba mate (Ilex paraguariensis) residue for the development of PLA-based bionanocomposite films. Ind. Crops Prod..

[19] Salas-Papayanopolos H., Morales-Cepeda A.B., Sanchez S., Lafleur P.G., Gomez I. (2017). Synergistic effect of silver nanoparticle content on the optical and thermo-mechanical properties of poly(l-lactic acid)/glycerol triacetate blends. Polym. Bull..

[20] Burgos N., Tolaguera D., Fiori S., Jiménez A. (2014). Synthesis and Characterization of Lactic Acid Oligomers: Evaluation of Performance as Poly(Lactic Acid) Plasticizers. J. Polym. Environ..

[21] Cicogna F., Coiai S., De Monte C., Spiniello R., Fiori S., Franceschi M., Braca F., Cinelli P., Fehri S.M.K., Lazzeri A. (2017). Poly(lactic acid) plasticized with low-molecular-weight polyesters: Structural, thermal and biodegradability features. Polym. Int..

[22] Burgos N., Martino V.P., Jiménez A. (2013). Characterization and ageing study of poly(lactic acid) films plasticized with oligomeric lactic acid. Polym. Degrad. Stab..

[23] Arrieta M.P., López J., López D., Kenny J.M., Peponi L. (2015). Development of flexible materials based on plasticized electrospun PLA-PHB blends: Structural, thermal, mechanical and disintegration properties. Eur. Polym. J..

[24] Khorshidi S., Solouk A., Mirzadeh H., Mazinani S., Lagaron J.M., Sharifi S., Ramakrishna S. (2016). A review of key challenges of electrospun scaffolds for tissue-engineering applications. J. Tissue Eng. Regen. Med..

[25] Basar A.O., Castro S., Torres-Giner S., Lagaron J.M., Turkoglu Sasmazel H. (2017). Novel poly(ε-caprolactone)/gelatin wound dressings prepared by emulsion electrospinning with controlled release capacity of Ketoprofen anti-inflammatory drug. Mater. Sci. Eng. C.

[26] (2020). Marina P Arrieta; Miguel Perdiguero; Stefano Fiori; José M Kenny; Laura Peponi Biodegradable electrospun PLA-PHB fibers plasticized with oligomeric lactic acid. Polym. Degrad. Stab..

[27] Leonés A., Mujica-Garcia A., Arrieta M.P., Salaris V., Lopez D., Kenny J.M., Peponi L. (2020). Organic and Inorganic PCL-Based Electrospun Fibers. Polymers.

[28] Leonés A., Peponi L., Lieblich M., Benavente R., Fiori S. (2020). In vitro degradation of plasticized PLA electrospun fiber mats: Morphological, thermal and crystalline evolution. Polymers.

[29] Rodríguez-Sánchez I.J., Fuenmayor C.A., Clavijo-Grimaldo D., Zuluaga-Domínguez C.M. (2021). Electrospinning of ultra-thin membranes with incorporation of antimicrobial agents for applications in active packaging: A review. Int. J. Polym. Mater. Polym. Biomater..

[30] Arrieta M.P., López J., López D., Kenny J.M., Peponi L. (2016). Effect of chitosan and catechin addition on the structural, thermal, mechanical and disintegration properties of plasticized electrospun PLA-PHB biocomposites. Polym. Degrad. Stab..

[31] Dziemidowicz K., Sang Q., Wu J., Zhang Z., Zhou F., Lagaron J.M., Mo X., Parker G.J.M., Yu D.-G., Zhu L.-M. (2021). Electrospinning for healthcare: Recent advancements. J. Mater. Chem. B.

[32] Mikos A.G., Temenoff J.S. (2000). Formation of highly porous biodegradable scaffolds for tissue engineering. Electron. J. Biotechnol..

[33] Zhu G., Zhang T., Chen M., Yao K., Huang X., Zhang B., Li Y., Liu J., Wang Y., Zhao Z. (2021). Bone physiological microenvironment and healing mechanism: Basis for future bone-tissue engineering scaffolds. Bioact. Mater..

[34] Leonés A., Salaris V., Mujica-Garcia A., Arrieta M.P., Lopez D., Lieblich M., Kenny J.M., Peponi L. (2021). PLA Electrospun Fibers Reinforced with Organic and Inorganic Nanoparticles: A Comparative Study. Molecules.

[35] Ferrández-Montero A., Lieblich M., González-Carrasco J.L., Benavente R., Lorenzo V., Detsch R., Boccaccini A.R., Ferrari B. (2019). Development of biocompatible and fully bioabsorbable PLA/Mg films for tissue regeneration applications. Acta Biomater..

[36] Boakye M., Rijal N., Adhikari U., Bhattarai N. (2015). Fabrication and Characterization of Electrospun PCL-MgO-Keratin-Based Composite Nanofibers for Biomedical Applications. Materials.

[37] De Silva R.T., Mantilaka M.M.M.G.P.G., Goh K.L., Ratnayake S.P., Amaratunga G.A.J., de Silva K.M.N. (2017). Magnesium Oxide Nanoparticles Reinforced Electrospun Alginate-Based Nanofibrous Scaffolds with Improved Physical Properties. Int. J. Biomater..

[38] Cramariuc B., Cramariuc R., Scarlet R., Manea L.R., Lupu I.G., Cramariuc O. (2013). Fiber diameter in electrospinning process. J. Electrostat..

[39] El-hadi A., Al-Jabri F. (2016). Influence of Electrospinning Parameters on Fiber Diameter and Mechanical Properties of Poly(3-Hydroxybutyrate) (PHB) and Polyanilines (PANI) Blends. Polymers.

[40] Martínez J.M.G., Collar E.P. (2020). On the combined effect of both the reinforcement and a waste based interfacial modifier on the matrix glass transition in ipp/a-pp-ppbma/mica composites. Polymers.

[41] García-Martínez J.-M., Collar E.P. (2020). The Role of a Succinyl Fluorescein-Succinic Anhydride Grafted Atactic Polypropylene on the Dynamic Mechanical Properties of Polypropylene/Polyamide-6 Blends at the Polypropylene Glass Transition. Polymers.

[42] Fisher R.A. (1960). The Desing of Experiments.

[43] Box G.E.P., Hunter W.G., Hunter J.S. (1978). Response surface methods. Statistics for Experimenters.

[44] Arrieta M.P., Gil A.L., Yusef M., Kenny J.M., Peponi L. (2020). Electrospinning of PCL-based blends: Processing optimization for their scalable production. Materials.

[45] Peponi L., Navarro-Baena I., Báez J.E., Kenny J.M., Marcos-Fernández A. (2012). Effect of the molecular weight on the crystallinity of PCL-b-PLLA di-block copolymers. Polymer.

[46] Manea L.R., Bertea A.-P., Nechita E., Popescu C.V. (2018). Mathematical Modeling of the Relation between Electrospun Nanofibers Characteristics and the Process Parameters. Electrospinning Method Used to Create Functional Nanocomposites Films.

[47] Piñeiro G., Perelman S., Guerschman J.P., Paruelo J.M. (2008). How to evaluate models: Observed vs. predicted or predicted vs. observed?. Ecol. Modell..

[48] Liu C., Shen J., Liao C.Z., Yeung K.W.K., Tjong S.C. (2018). Novel electrospun polyvinylidene fluoride-graphene oxide-silver nanocomposite membranes with protein and bacterial antifouling characteristics. Express Polym. Lett..

[49] Kathrein H., Freund F. (1983). Electrical conductivity of magnesium oxide single crystal below 1200 K. J. Phys. Chem. Solids.

[50] Habeeb M.A., Hamza R.S.A. (2018). Synthesis of (Polymer blend-MgO) nanocomposites and studying electrical properties for piezoelectric application. Indones. J. Electr. Eng. Inform..

[51] Khan R.A.A., Qi H.-K., Huang J.-H., Luo M.-B. (2021). A simulation study on the effect of nanoparticle size on the glass transition temperature of polymer nanocomposites. Soft Matter.

[52] Serenko O.A., Roldughin V.I., Askadskii A.D., Serkova E.S., Strashnov P.V., Shifrina Z.B. (2017). The effect of size and concentration of nanoparticles on the glass transition temperature of polymer nanocomposites. RSC Adv..

[53] Papageorgiou G.Z., Achilias D.S., Bikiaris D.N., Karayannidis G.P. (2005). Crystallization kinetics and nucleation activity of filler in polypropylene/surface-treated SiO2 nanocomposites. Thermochim. Acta.

[54] Shi X., Zhang G., Phuong T.V., Lazzeri A. (2015). Synergistic effects of nucleating agents and plasticizers on the crystallization behavior of Poly(lactic acid). Molecules.

[55] Clarkson C.M., El Awad Azrak S.M., Schueneman G.T., Snyder J.F., Youngblood J.P. (2020). Crystallization kinetics and morphology of small concentrations of cellulose nanofibrils (CNFs) and cellulose nanocrystals (CNCs) melt-compounded into poly(lactic acid) (PLA) with plasticizer. Polymer.

